# Pain flashbacks: The role of intrusive pain symptoms in posttraumatic chronic pain

**DOI:** 10.1192/j.eurpsy.2023.307

**Published:** 2023-07-19

**Authors:** N. Tsur, C. Katz

**Affiliations:** Tel Aviv University, Tel Aviv, Israel

## Abstract

**Introduction:**

Findings demonstrate the high comorbidity of posttraumatic stress disorder (PTSD) and chronic pain following exposure to trauma. In exposure to child abuse (CA) in particular, findings imply that CA survivors are at a higher risk of suffering from chronic pain. However, the underlying mechanisms of these processes are yet to be uncovered.

**Objectives:**

This study examined a new mechanism pertaining to the potential role of intrusive pain flashbacks for explaining the link between CA, C/PTSD, and chronic pain following interpersonal trauma.

**Methods:**

A community sample of 430 women (Sample A), and a sample of 164 women who were exposed to CA (Sample B) completed questionnaires assessing pain flashbacks, CA, C/PTSD symptoms, the experience of pain during the trauma, and chronic pain.

**Results:**

The findings showed that 8.9% of Sample A (N = 36), and 23.1% of Sample B (N = 37) reported experiencing pain flashbacks. In both samples, participants who experienced pain flashbacks reported more severe C/PTSD (*p*<0.001), compared to participants who experienced flashbacks without pain and those who did not experience pain flashbacks. Participants who experienced pain flashbacks reported more pain during CA (*p =* 0.001), which corresponded with the pain flashbacks areas (Figure 1). Finally, pain flashbacks were correlated with a higher risk of suffering from chronic pain in Sample B (*p*=0.002).

**Image:**

**Figure:**
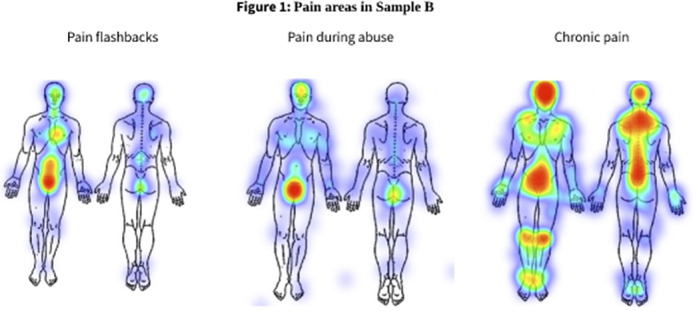

**Conclusions:**

The findings of this study reveal that pain flashbacks are associated with more rampant CA and are linked to greater psychopathology. The findings call for further investigation of the role of pain flashbacks in explaining the link between exposure to trauma, C/PTSD and later chronic pain.

**Disclosure of Interest:**

None Declared

